# Recurrent appendicitis of vermiform appendix after a prior appendectomy: A case report and review of the literature

**DOI:** 10.1016/j.amsu.2022.103603

**Published:** 2022-04-13

**Authors:** Talal Almas, Vikneswaran Raj Nagarajan, Danyal Ahmed, Muneeb Ullah, Mohammed Ali Ashary, Mert Oruk, Arsalan Khan, Kiran Amin, Uzair Malik, Joshua Ramjohn, Helen Huang, Ali Rifai, Ahlam Alzahrani, Nagi Alqallaf, Sood Alsairefi, Yeoreum Summer Hur, Anhad Bhullar, Khadeer Abdulkarim, Eissa Alwheibi, Mhmod Kadom, Aaisha Alshabibi, Adil Shafi, Faisal Murad, Emad Mansoor

**Affiliations:** a RCSI University of Medicine and Health Sciences, Dublin, Ireland; b Department of Surgery, Maroof International Hospital, Islamabad, Pakistan; c St. George's University School of Medicine, London, UK; d Sligo University Hospital, Sligo, Ireland; e Division of Gastroenterology and Liver Disease, University Hospitals Cleveland Medical Center, Case Western Reserve University, Cleveland, OH, USA

## Abstract

**Introduction:**

Acute appendicitis is one of the leading causes of acute abdominal pain and surgical emergency. Stump appendicitis is a known complication of appendectomy whereby a retained appendiceal tip serves as a nidus for recurrent bouts of inflammation. Nevertheless, full-blown appendicitis of the vermiform appendix after a prior appendectomy remains a diagnostic conundrum.

**Case presentation:**

A 45-year-old woman presented with a six-month history of right iliac fossa pain. Pertinently, she had undergone a prior open appendectomy twelve years ago. Further investigative workup revealed full-blown appendicitis, which was not attributable to a retained appendiceal stump. A subsequent laparoscopic appendectomy was performed, and the resultant specimen was sent for further evaluation, confirming the diagnosis of recurrent appendicitis.

**Clinical discussion:**

Acute appendicitis is one of the most common life-threatening abdominal surgical emergencies worldwide, with 300000 appendectomies performed annually in the United States alone. Stump and chronic appendicitis are two separate and exceedingly rare clinical entities that may present simultaneously and develop serious complications unless promptly recognized and appropriately managed. The present paper prompts the clinicians to distinguish amongst the two at the initial surgery in order to thwart further exacerbations.

**Conclusion:**

While stump appendicitis is a rare but well-characterized complication of a prior appendectomy, full-blown appendicitis of vermiform appendix remains elusive. It is therefore imperative to distinguish between a duplicated and a recurrent appendix at the initial operative procedure to facilitate optimal patient management.

## Highlights

• Recurrent appendicitis in the aftermath of a prior appendectomy remains an under-recognized cause of acute right iliac fossa pain.
• While stump appendicitis is a well-characterised complication of laparoscopic or open appendectomy, full-blown appendicitis of a regrown appendix continues to be an elusive clinicopathological phenomena.
• It remains crucial to distinguish between a duplicated appendix and a regrown appendix in the wake of an appendectomy to better inform the debate on its optimal medical or surgical management.
• Correctly identifying the appendiceal base during surgery and ensuring a post-resection stump length of no more than 3 mm can preclude the possibility of stump appendicitis.
• Surgeons should be cognizant of this anatomical aberration and should therefore exclude appendiceal duplication at the time of the index surgery.

## 1 Introduction

Acute appendicitis remains a leading cause of acute right iliac fossa pain, often presenting as a life-threatening surgical emergency warranting prompt surgical treatment. In the United States alone, 300000 appendectomies are performed annually, with the rates soaring even higher in other regions globally [1,2]. Acute appendicitis is thought to result from a myriad of etiologies including obstruction of the appendiceal lumen, bacterial colonization, growth, inflammation, bowel ischemia, or bowel perforation [3]. The definitive pathophysiology is poorly understood and poses a challenge in diagnosing acute appendicitis pre-operatively. The current diagnostic algorithm of acute appendicitis has been based on initial clinical judgement, followed by imaging and laboratory tests. Acute abdomen symptoms such as right lower quadrant (RLQ) pain, rigidity, and periumbilical pain radiation to the right iliac fossa (RIF) are classic for acute appendicitis [4,5]. In current practice, clinical scoring scales are utilised with supporting laboratory tests and are widely advocated to stratify the risk of acute appendicitis. The most widely accepted scaling systems that guide the management of acute appendicitis consist of the Alvarado score, Pediatric appendicitis Score, or the Appendicitis Inflammatory Response score [[6], [7], [8], [9], [10]]. The integration of point-of-care ultrasonography has improved the diagnostic accuracy of acute appendicitis and complements the scoring systems [11]. Depending on their disease severity, patients will be managed medically or surgically and can undergo definitive treatment through either open or laparoscopic appendectomy. Though a standard treatment for appendicitis, more commonly reported complications include wound infection, abscess, perforation, and sepsis [12,13]. These risks equate to longer hospital stays and extended hospital stays which can hinder the recovery of patients. However, an underreported and serious complication of appendectomies includes recurrent or chronic appendicitis, which is often misdiagnosed in patients surgically treated for acute appendicitis in the past.

Stump appendicitis, although uncommon, is a relevant and devastating complication of both open and laparoscopic appendectomies. The residual appendiceal tissue after the procedure can become repeatedly inflamed and leaves a stump, predisposing patients to recurrent appendicitis. Although there is no reported incidence, it is presumably thought to be between 0.002% and 0.15% occurring anytime between 5 weeks and 17.5 years postoperatively [4,5]. Despite the low incidence, this still represents a commonly overlooked cause of RIF pain in patients with prior appendectomies and is usually not considered a preliminary diagnosis [6]. An appendiceal stump poses a dilemma to clinicians and is commonly associated with a late diagnosis if one is unaware of the uncommon clinical presentations that distinguish a stump from acute appendicitis [7]. Despite newer imaging modalities and technology, pre-operative diagnosis of appendiceal stumps remains a clinical conundrum and is associated with an increased risk of perforation due to a delay in referral and management [8]. The etiology of stump appendicitis is unclear but thought to be predisposed by medical and surgical factors following a previous appendectomy. However, another diagnostic dilemma leading to recurrent appendicitis is the possibility of an underlying duplicated appendix, which may be overlooked during initial preoperative and peri-operative workup. This congenital anomaly was first reported in 1892 and since then, has been reported as an incidental finding that was commonly “missed” in pre-operative investigations [9,10]. The reported incidence rate of the duplicated appendix is exceedingly low, hovering around 0.004% [7]. However, thorough exploration of the caecum during laparotomy can avoid delayed diagnosis, serious complications, and medicolegal consequences in the future [8].

Herein, we chronicle the case of a 45-year-old female patient presenting with a constellation of symptoms typical of acute appendicitis on a background history of prior appendectomy. Second laparoscopic appendectomy confirmed a single appendectomy scar in the colon, reaffirming the diagnosis of a regrown, rather than a duplicated, vermiform appendix. The present paper was reported in accordance with the SCARE guidelines [9].

## 2 Case presentation

A 45-year-old woman presented to the hospital with a six month history of intermittent right iliac fossa (RIF) pain. The patient denied experiencing nausea, vomiting, urinary or bowel symptoms and had undergone open appendectomy twelve years prior to the current presentation. Pertinently, investigative workup at the time had excluded the possibility of appendiceal duplication, and subsequent open appendectomy further reaffirmed this notion. Upon physical examination, the patient demonstrated mild tenderness in the RIF with positive rebound tenderness. The rovsing's sign was negative at the time, with no evidence of rigidity, guarding, or peritonitis. The patient also had a well healed grid-iron scar in the RIF from her previous open appendectomy.

**Fig. 1:**
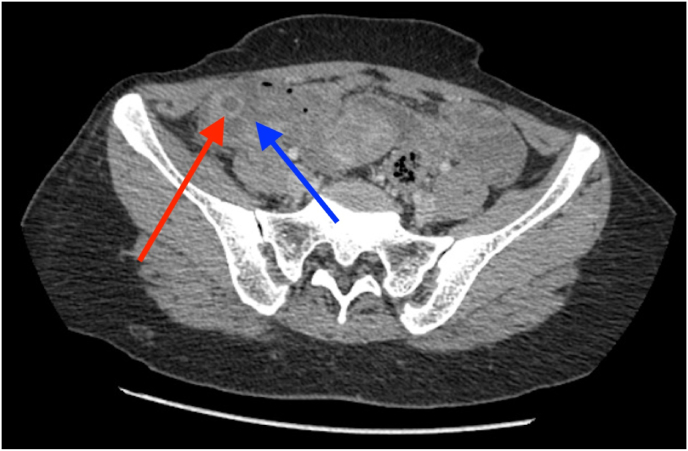
Axial view.

**Fig. 2:**
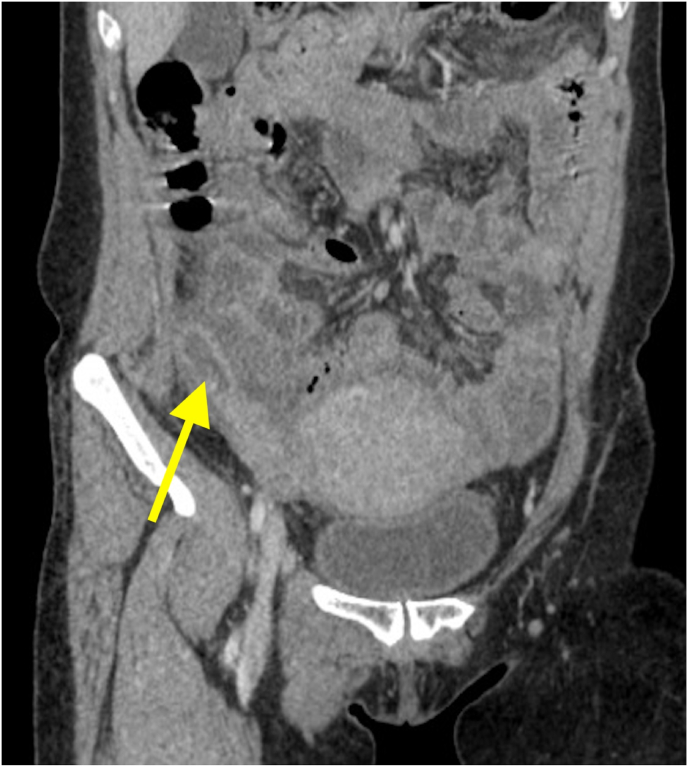
A coronal non-contrast CT scan.

Ultrasound imaging of the RIF showed a complex ovarian mass and further non-contrast computed tomography (CT) scans (Fig. 1 and Fig. 2) revealed enhancement and thickening at the splenic flexure as well as a tubular, blind-ended structure in the RIF resembling an appendix which was noted to be dilated, fluid-filled with a caliber of 10 mm. The structure showed thickened walls with surrounding mild free fluid present. Given the presence of a complex ovarian mass, CA-125 levels were obtained and were borderline raised. However, a subsequent colonoscopy and barium follow-through yielded normal results and effectively excluded the presence of an ovarian malignancy.

Non-contrast abdominal CT demonstrating an inflamed appendix (red arrow) and periappendiceal collection and stranding (blue arrow).

The inflamed appendix can be seen (yellow arrow), with periappendiceal stranding and fibrosis.

**Fig. 3:**
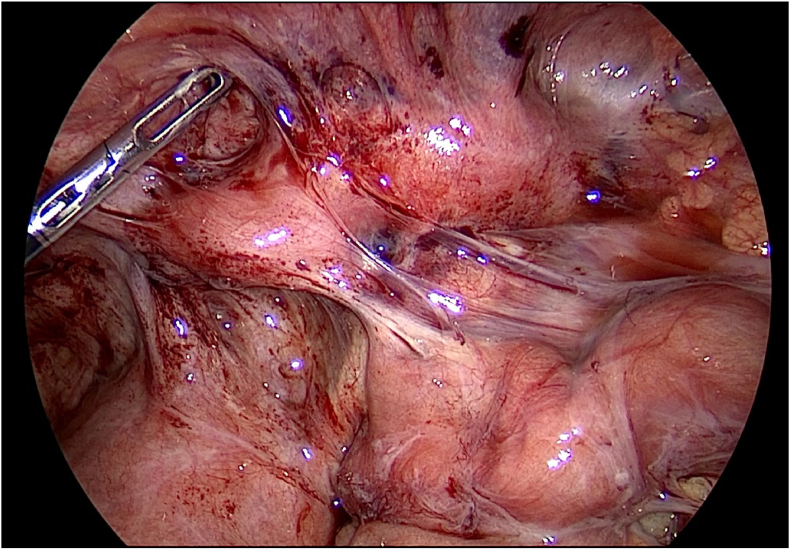
Inflamed appendix can be visualized along with surrounding adhesions.

Non-contrast CT scanning of the abdomen and the RIF divulged dilated thick walled, fluid-filled tubular structure in the RIF with mild free fluid and lymphadenopathy with the largest lymph node measuring 14 × 9.2 mm in size, likely indicative of acute/subacute appendicitis. Another possible differential for the imaging finding is a simple left adnexal cyst, which was also evaluated (Fig. 3).

Thereafter, the patient was admitted and prepared for a diagnostic laparoscopy and laparoscopic appendectomy. Per-operatively, an acute inflamed appendix filled with pus was seen adherent to the lateral wall near the iliac vessels (Fig. 1, Fig. 2, Fig. 3).

**Fig. 4:**
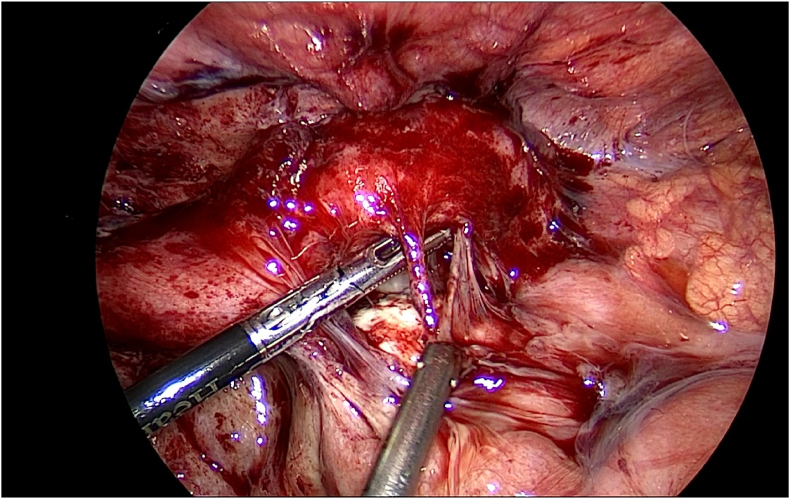
Further evidence of adhesions, indicative of chronic infections, can be seen per-operatively.

Fig. 4 further demonstrates the presence of diffuse adhesions.

**Fig. 5:**
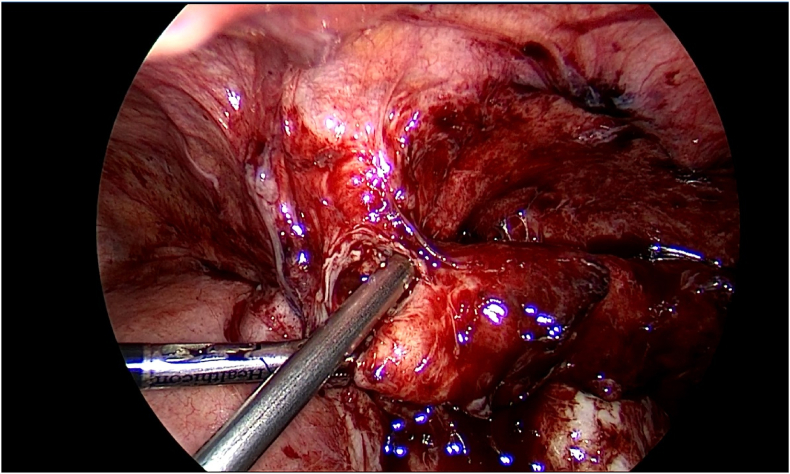
Blunt dissection at the appendiceal tip.

Interestingly, laparoscopy divulged evidence of blunt dissection at the appendiceal tip (Fig. 5).

**Fig. 6:**
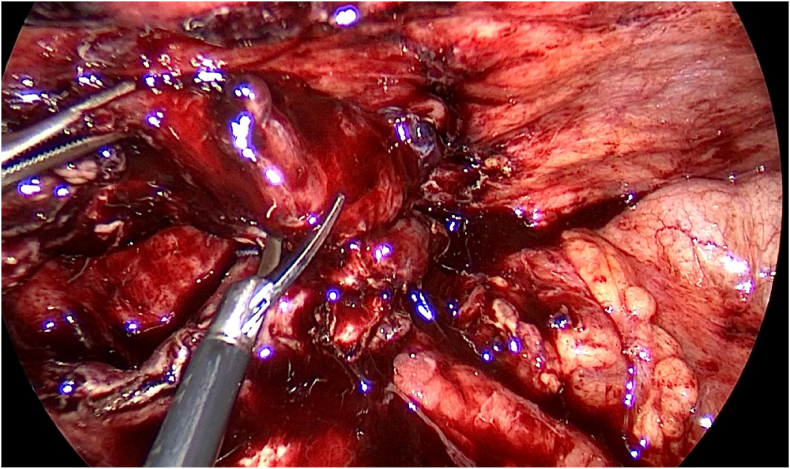
Resecting the appendix after ligation at the appendiceal base.

The tip of the appendix was adherent to the round ligament and 20 mL of reactionary fluid was found in the Pouch of Douglas. A simple left ovarian cyst measuring 3 × 2 cm was also incidentally found. The fallopian tubes, ovaries and uterus were unremarkable, and per-operative findings pragmatically precluded the presence of a concomitant malignant process. Intraoperatively, the appendix was resected after ligating the bade (Fig. 6).

**Fig. 7:**
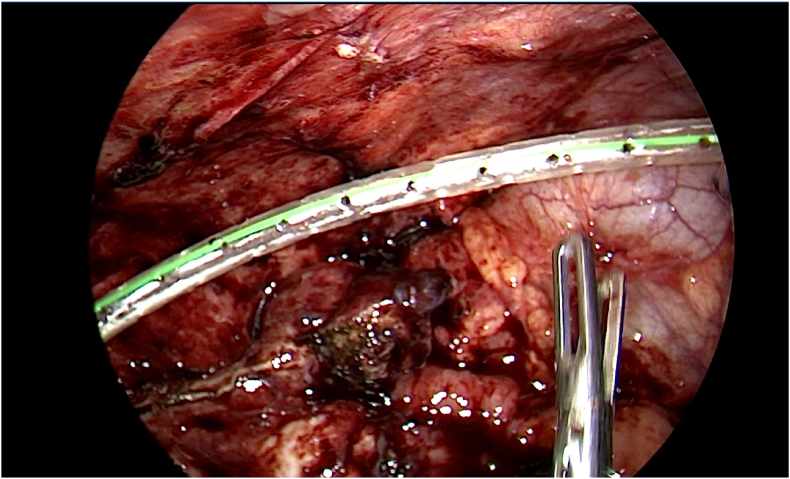
Intraoperative laparoscopy image demonstrating drain placement at the stump site.

Finally, a drain was placed at the stump site (Fig. 7).

The patient was discharged in the evening and postoperatively she recovered well with no postoperative complications. Histopathological analysis of the appendix showed extensive fibrosis, and moderate acute and chronic inflammation with serositis, strongly indicating a full-blown inflammatory process involving the appendix. There was no evidence of malignancy or parasitic infection. The patient continues to do well to date, with no further exacerbations.

## 3 Discussion

We highlight a unique case presenting with 6-month history of intermittent appendiceal colic occurring 12 years post-open appendectomy. To further clinical understanding of atypical appendicitis, this discussion will address the different forms discovered in our review of the literature and attempt to delineate some of the uncertainty around this topic. While acute appendicitis is well-recognized and a top differential for right lower quadrant pain there is far less known regarding atypical presentations of appendicitis such as chronic and recurrent stump appendicitis and indeed, little concordance in the literature regarding their definitions [10].

Stump and chronic appendicitis are two separate and exceedingly rare clinical entities that may present simultaneously, as in our case, and develop serious complications unless promptly recognized and appropriately managed. In the literature, both are sometimes referred to as recurrent appendicitis [11,12] however in this paper we refer to stump and recurrent appendicitis interchangeably. Stump appendicitis occurs due to inflammation of the residual appendiceal stump occurring anywhere from several days to years after the initial appendectomy [13]. The disease course of stump appendicitis can mirror chronic appendiceal inflammation, as both present with recurrent symptoms requiring additional investigations. Moreover, similar clinical presentations of stump appendicitis to primary acute appendicitis can create difficulty in its diagnosis and prolong immediate treatment.

There is yet another possible differential of recurrent appendicitis occurring in a duplicated appendix after prior appendectomy [14]. Duplicated appendices occur with an estimated incidence of 0.004% and to date, there are 4 main types of appendiceal duplications known according to Cave–Wallbridge classifications; Type A, B, C and D. Most pertinent to our case, Type B duplications involving duplicated appendices on either side of the ileocecal valve (B1) or a normally located appendix followed by a second retrocaecal appendix (B2) were distinct diagnostic possibilities [14]. However, the initial resection was conducted upon open appendectomy and there was only a singular incision site left on repeat surgery. As such, the possibility of appendiceal duplication was ruled out from our case and we maintain that this was indeed a rare case of stump appendicitis.

The treatment for appendicitis remains appendectomy performed either open or laparoscopically. Despite the reduced visual field, laparoscopic appendectomies have several benefits including lower rates of complications, faster postoperative recovery [15] and a previous literature review found only 34% of recurrent stump appendicitis cases were followed by initial laparoscopic appendectomy [4].

Atypical appendicitis, especially following previous appendectomy, is often misdiagnosed and conservative treatment with antibiotics can delay, mask or transiently resolve symptoms only for patients to return on repeat onset of RIF pain [3]. Previous studies have shown it is often misdiagnosed as constipation and gastroenteritis [16] and delayed diagnosis has been associated with perforations, small bowel obstruction, abdominal abscesses and even adenocarcinoma of the residual stump [17].

In order to better elucidate the aetiology underlying the recurrent appendicitis observed in the present case, we conducted a literature search using the digital databases (PubMed/MEDLINE, CINAHL, and Web of Science) to search for relevant material and articles reporting any cases and advancements in the diagnosis and management of recurrent full-blown appendicitis. The literature search was conducted using the terms(s): “recurrent appendicitis” AND “vermiform appendix” OR “stump appendicitis” OR “appendiceal duplication”. The symptomatology, imaging findings, treatment employed, and the follow-up are delineated by Table 1 below [[19], [20], [21], [22], [23], [24], [25], [26], [27], [28], [29], [30], [31], [32], [33], [34], [35], [36], [37], [38], [39], [40], [41], [42], [43], [44], [45], [46], [47], [48], [49], [50], [51], [52], [53], [54], [55], [56], [57], [58], [59], [60], [61]].

**Table 1:** Literature Review on Stump Appendicitis 1945–2021* Literature review was conducted using search terms “stump appendicitis” and “recurrent stump appendicitis” on PUBMED.30 cases published in 2011 or newer with available full text available were included in addition to 40 cases extracted from a prior literature review [13].

Author	Year	Age	Sex	Initial Treatment	Presenting Complaint	Interval	Ds	Repeat Treatment	Stump Length
Rose [19]	1945	23	M	Open	NR	1 year	NR	Open	5.1cm
Rose [19]	1945	40	M	Open	NR	2 year	NR	Open	5.1cm
Baumgardner [20]	1949	55	M	Open	RIF	2 months	NR	Open	NR
Siegel [21]	1954	51	F	Open	RIF	23 years	NR	Open	1.5cm
Greene [22]	1958	27	F	Open	RIF	12 years	NR	Open	NR
Greene [22]	1958	42	F	Open	ABD	16 years	NR	Open	NR
Greene [22]	1958	53	F	Open	RIF	20 years	NR	Open	NR
Harris [23]	1989	26	M	Open	RIF	10 years	CT	Open	NR
Feigin [24]	1993	26	M	Open	ABD	1 years	NR	Open	NR
Devereaux [25]	1994	49	M	Lap	RIF	2 months	NR	Open	2cm
Thomas [26]	1994	53	F	Open	RIF	21 years	CT	Open	NR
Wright [27]	1994	35	M	Lap	RIF	2 months	NR	Open	4.5cm
Wright [27]	1994	48	M	Lap	RIF	8 months	CT	Open	4.0cm
Greenberg [28]	1996	31	M	Lap	RIF	4 months	CT	Open	3.5cm
Milne [29]	1996	25	M	Lap	ABD	18 months	NR	Open	3.2cm
Walsh [30]	1997	72	F	Lap	ABD	5 months	Xray	Open	2.5cm
Erzurum [31]	1997	11	F	Open	RIF	8 months	CT	Open	3.5cm
Rao [32]	1998	39	F	Open	ABD	34 years	CT	Open	NR
Mangi [33]	2000	43	F	Open	RIF	40 years	CT	Open	0.5cm
Mangi [33]	2000	64	F	Open	RIF	NR	BE	Open	0.6cm
Baldisserotto [34]	2000	13	F	Open	RIF	2 months	US	Lap	2cm
Gupta [35]	2000	11	M	Open	RIF	1 year	CT	Open	4.5cm
Nahon [36]	2002	33	M	Open	RIF	18 years	Colonoscopy	Open	NR
Chikamori [37]	2002	24	M	Lap	ABD	4 days	US	Lap	7mm
Durgun [38]	2003	68	F	Open	ABD	8 months	NR	Open	3cm
Watkins [39]	2004	63	F	Lap	RIF	9 months	CT	Lap	5.5cm
De U [40]	2004	26	F’	Open	RIF	1 year	NR	Open	NR
Aschkenasy [41]	2005	27	M	Open	RIF	25 years	CT	Open	NR
Roche-Nagle [42]	2005	35	M	NR	RIF	NR	CT	Open	3–4cm
Shin [43]	2005	41	M	Lap	RIF	NR	CT	Lap	6.5cm
Burt [44]	2005	27	M	Open	RIF	NR	CT	Open	NR
Liang [4]	2006	32	F	Lap	RIF	5 months	CT	Lap	4cm
Uludag [45]	2006	47	M	Open	RIF	20 years	CT	Open	2cm
Waseem [46]	2008	15	M	Lap	ABD	2 years	CT	Open	6mm
Leff [16]	2010	33	F	Lap	RIF	2 weeks	CT	App	NR
Leff [16]	2010	24	M	Lap	ABD	7 months	CT	Lap	NR
O'Leary [47]	2010	43	M	Open	RIF	10 years	US	Open	2.5cm
Tang [48]	2011	14	M	Open	ABD	5 years	CT	Open	3cm
Tang [48]	2011	11	M	Open	NR	2 months	CT	Open	NR
Tang [48]	2011	13	F	Open	ABD	10 months	CT	Open	4cm
Parameshwarappa [49]	2011	18	M	Lap	ABD	1 year	CT	Open	NR
Awe [50]	2013	25	F	Lap	ABD	4.5 months	US/CT	Lap	NR
Hashmi [51]	2013	20	M	Open	RIF, vomiting	10 years	CT	IV Antibiotics	NR
Minguez [52]	2013	67	W	Open	ABD	7 months	CT	Lap	2 cm
Minguez [52]	2013	30	W	Open	RIF	6 months	US	Lap	3 cm
Minguez [52]	2013	24	M	Lap	ABD	1 day	US	Lap	NR
Chamorro [53]	2013	15	M	Open	RIF	2 years	US	NR	28mm
Chamorro [53]	2013	38	M	Open	RIF	20+ years	US/CT	Lap	6cm
Chamorro [53]	2013	30	M	Open	RIF	18 years	CT	Lap	7cm
Chamorro [53]	2013	24	M	Open	RIF	5 years	US	Lap	18mm
Artul [54]	2014	20	M	App	RIF, vomiting	2 months	CT	Antibiotics	18mm
Zachariah [55]	2014	58	F	Lap	ABD/RIF, vomiting	25 years	US/CT	Lap	2cm
Constantin [56]	2014	26	F	Lap	ABD	2 months	US	NR	4cm
Constantin [56]	2014	40	M	Open	RIF	15 years	US	NR	NR
Rios [57]	2015	33	F	App	ABD	6 months	CT	NR	NR
Rios [57]	2015	34	F	App	ABD	5 years	CT	NR	NR
Chandran [58]	2015	63	M	Lap	RIF	2 years	CT	NR	NR
Cobb [59]	2015	63	M	Lap	ABD, RIF	2 years	CT	App	NR
Cifti [60]	2015	17	M	Lap	ABD, RIF	6 months	US	Lap	3 cm
Maurice [61]	2016	31	M	Lap	RIF	5 years	CT	Lap cecectomy	2cm
Ekici [62]	2016	26	M	Open	ABD, RIF	6 months	CT	Lap	5 cm
Shah [63]	2017	35	F	Open	RIF	4.5 months	US	Lap	NR
Al Shehri [18]	2017	39	M	Open	RIF	14–16 years	CT	Lap	NR
Giwa [64]	2018	32	M	App x2	RIF	7 days, 5 days	CT	App	1.3cm
Geraci [6]	2019	54	F	Lap	ABD	46 years	CT	Lap	24mm
Boardman [65]	2019	50	M	Lap	ABD	1 year	CT	Lap	NR
Burbano [66]	2020	49	M	Open	RIF, vomiting	31+ years	CT	Lap	1.3cm
Mizuta [67]	2020	32	F	Lap	ABD	2.5 years	CT	Lap	NR
Castaneda [68]	2021	38	F	Lap	ABD	5 years	CT	Lap	14.25mm
Hadrich [69]	2021	30	F	App	ABD, fever	10 months	CT	Lap cecectomy	2cm

Open = open appendectomy, Lap = laparoscopic appendectomy App = unknown appendectomy NR = not reported US = ultrasound CT = computerized tomography ABD = diffuse abdominal pain RIF = right iliac fossa pain.

While most studies in our review were able to identify stump appendicitis using CT imaging (Table 1), some resorted to diagnostic laparotomies. In one case where conservative management was repeatedly pursued, the patient experienced 3 episodes of undiagnosed chronic recurrent stump appendicitis until eventual diagnostic laparotomy and subsequent appendiceal stump resection resolved all symptoms [18]. As such, we recommend clinicians consider atypical appendicitis in any patient presenting with RLQ pain regardless of prior appendectomy and withhold antibiotics in favour of full workup including radiological investigation followed by laparoscopic exploration if needed. In addition, we reiterate prior recommendations emphasising the importance of correct identification of the appendiceal base and ensuring post-resection stump length of no more than 3 mm for any resected appendices ≤6.5cm in length to avoid future stump appendicitis [13].

### 3.1 Limitations

The overarching limitation in our cases was, upon index surgery, the presence of a duplicated appendix was not precluded. As such, it could be the case that the recurrent appendicitis could be afflicting the duplicated, rather than a regrown, appendix. In order to curb this possibility, surgeons should be cognizant of this anatomical aberration and should therefore exclude appendiceal duplication at the time of the index surgery.

## 4 Conclusion

While stump appendicitis is a rare but well-characterised complication of a prior appendectomy, full-blown appendicitis of vermiform appendix remains elusive. It is therefore imperative to distinguish between a duplicated and a recurrent appendix at the initial index operative procedure to facilitate optimal patient management. Correctly identifying the appendiceal base during surgery and ensuring a post-resection stump length of no more than 3 mm can preclude the possibility of stump appendicitis. Furthermore, surgical incision sites should be evaluated during repeated appendectomies to distinguish an appendiceal duplication from stump appendicitis to better dictate optimal patient management.

## Disclosure

None.

## Sources of funding

NA.

## Ethical approval

NA.

## Consent

Written informed consent was obtained from the patient for publication of this case report and accompanying images. A copy of the written consent is available for review by the Editor-in-Chief of this journal on request.

## Author contribution

TA, VRN, DA, MU, MAA, MO: conceived the idea, designed the study, and drafted the manuscript. HH, KA, UM, JR, AR, KA: conducted literature search and created the illustrations. AA, AA, NA, SA, YSH, HH, AB: revised the manuscript critically and refined the table, literature search results, and addressed reviewer comments. AS, FM, EM, EA, MK: revised the final version of the manuscript critically and gave the final approval.

## Registration of research studies

1. Name of the registry: NA.

2. Unique Identifying number or registration ID: NA.

3. Hyperlink to your specific registration (must be publicly accessible and will be checked): NA.

## Guarantor

Talal Almas.

RCSI University of Medicine and Health Sciences.

123 St. Stephen's Green.

Dublin 2, Ireland.

Talalalmas.almas@gmail.com.

## Provenance and peer-review

Not commissioned, externally peer-reviewed.

## Declaration of competing interest

NA.
